# 4-Bromo­thio­benzamide

**DOI:** 10.1107/S1600536809018273

**Published:** 2009-05-20

**Authors:** Mahmood-ul-Hassan Khan, Shahid Hameed, Tashfeen Akhtar, Jason D. Masuda

**Affiliations:** a Department of Chemistry, Quaid-i-Azam University, Islamabad 45320, Pakistan; b Department of Chemistry, Saint Mary’s University, Halifax, Nova Scotia, Canada B3H 3C3

## Abstract

The title compound, C_7_H_6_BrNS, crystallizes with two mol­ecules in the asymmetric unit. The dihedral angles between the aromatic ring and the thio­amide fragment are 23.6 (4) and 20.5 (3)° in the two mol­ecules. In the crystal, there are inter­molecular N—H⋯S hydrogen-bonding inter­actions between the amine group and the S atoms.

## Related literature

For the uses of thio­amides, see: Akhtar *et al.* (2006 ▶, 2007 ▶, 2008 ▶); Jagodzinski *et al.* (2003 ▶). For the biological activity of thio­amides, see: Wei *et al.* (2006 ▶); Klimesova *et al.* (1999 ▶). For the synthesis of thio­amides, see: Kaboudin *et al.* (2006 ▶); Cava *et al.* (1985 ▶). For related crystal structures, see: Khan *et al.* (2009 ▶); Jian *et al.* (2006 ▶); Manaka & Sato (2005 ▶).
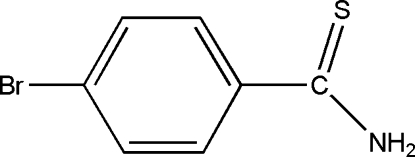


## Experimental

### 

#### Crystal data

C_7_H_6_BrNS
*M* *_r_* = 216.10
Monoclinic, 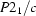

*a* = 19.6325 (11) Å
*b* = 10.6101 (6) Å
*c* = 7.8859 (5) Å
β = 100.078 (1)°
*V* = 1617.31 (16) Å^3^
*Z* = 8
Mo *K*α radiation
μ = 5.26 mm^−1^
*T* = 296 K
0.21 × 0.17 × 0.09 mm

#### Data collection

Bruker APEXII CCD diffractometer
Absorption correction: multi-scan (*SADABS*; Bruker, 2008 ▶) *T*_min_ = 0.384, *T*_max_ = 0.620
12968 measured reflections
3911 independent reflections
2706 reflections with *I* > 2σ(*I*)
*R*_int_ = 0.025

#### Refinement

*R*[*F* ^2^ > 2σ(*F* ^2^)] = 0.035
*wR*(*F* ^2^) = 0.087
*S* = 1.03
3911 reflections
181 parameters
H-atom parameters constrained
Δρ_max_ = 0.82 e Å^−3^
Δρ_min_ = −0.78 e Å^−3^

### 

Data collection: *APEX2* (Bruker, 2008 ▶); cell refinement: *SAINT* (Bruker, 2008 ▶); data reduction: *SAINT*; program(s) used to solve structure: *SHELXS97* (Sheldrick, 2008 ▶); program(s) used to refine structure: *SHELXL97* (Sheldrick, 2008 ▶); molecular graphics: *ORTEP-3 for Windows* (Farrugia, 1997 ▶); software used to prepare material for publication: *SHELXTL* (Sheldrick, 2008 ▶).

## Supplementary Material

Crystal structure: contains datablocks I, global. DOI: 10.1107/S1600536809018273/bt2956sup1.cif

Structure factors: contains datablocks I. DOI: 10.1107/S1600536809018273/bt2956Isup2.hkl

Additional supplementary materials:  crystallographic information; 3D view; checkCIF report

## Figures and Tables

**Table 1:** Hydrogen-bond geometry (Å, °)

*D*—H⋯*A*	*D*—H	H⋯*A*	*D*⋯*A*	*D*—H⋯*A*
N2—H2*A*⋯S2^i^	0.86	2.73	3.583 (2)	172
N2—H2*B*⋯S1^ii^	0.86	2.65	3.500 (2)	173
N1—H1*A*⋯S1^iii^	0.86	2.78	3.605 (3)	160
N1—H1*B*⋯S2^ii^	0.86	2.71	3.523 (2)	158

Symmetry codes: (i) 
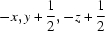
; (ii) 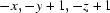; (iii) 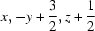.

**Fig. 1.**
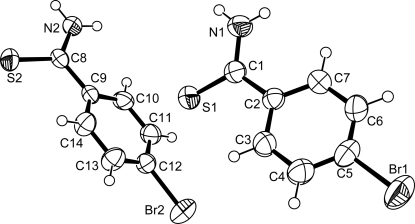
Molecular structure of 4-bromobenzothioamide showing displacement ellipsoids at the 50% probability level (for non-H atoms).

**Fig. 2.**
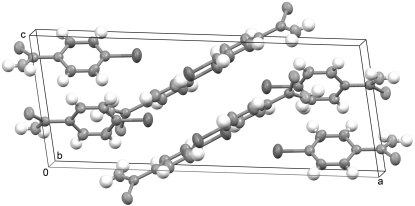
Packing diagram of 4-bromobenzothioamide. Displacement ellipsoids are shown at the 50% probability level (for non-H atoms).

## supplementary crystallographic information

### Comment

Thioamides are biologically active compounds, possessing a wide spectrum of activities (Klimesova *et al.*, 1999; Wei *et al.*, 2006). They have enormous practical and synthetic applicability and their importance and impact as synthetic intermediates is continuously growing (Jagodzinski *et al.*, 2003). Thioamides are generally synthesized using Lawesson's reagent (Cava *et al.*, 1985) or phosphorus penta sulfide (Kaboudin *et al.*, 2006). In this article, we wish to report the crystal structure of 4-bromobenzothioamide, which was synthesized by treating 4-bromobenzonitrile with 70% sodium hydrogen sulfide hydrate and magnesium chloride hexahydrate (Manaka & Sato, 2005) in continuation of our previous work on the synthesis and biological screenings of five membered heterocycles (Akhtar *et al.*, 2006, 2007, 2008).

The hydrogen bonding interactions between the nitrogen and sulfur atoms (3.500 (2)Å to 3.605 (3) Å) are in the range of those seen in *p*-trifluoromethylbenzothioamide where the corresponding interactions are between 3.3735Å and 3.5133Å (Jian *et al.*, 2006) and in the analogus chloride compound where the N···S distances are 3.3769 (15)Å and 3.4527 (15)Å (Khan *et al.*, 2009).

### Experimental

The slurry of 70% sodium hydrogen sulfide hydrate (21.98 mmol) and magnesium chloride hexahydrate (10.99 mmol) was prepared in DMF (40 mL). 4-Bromobenzonitrile (11.0 mmol) was added to the slurry and the mixture stirred at room temperature for 2 h. The resulting mixture was poured into water (100 mL) and the precipitated solid collected by filtration. The product obtained was resuspended in 1 N HCl (50 ml), stirred for another 30 min, filtered and washed with excess water. The recrystallization of the product from chloroform afforded the crystals of 4-bromobenzothioamide suitable for X-ray analysis.

### Refinement

The hydrogen atoms were placed in geometrically idealized positions of 0.93Å (aromatic C—H) and 0.86Å (amide N—H) and constrained to ride on the parent atom with *U*_iso_(H) = 1.2 UEq(*c*) for aromatic and amide protons.

### Crystal data

**Table:**

C_7_H_6_BrNS	*F*(000) = 848
*M**_r_* = 216.10	*D*_x_ = 1.775 Mg m^−^^3^
Monoclinic, *P*2_1_/*c*	Mo *K*α radiation, λ = 0.71073 Å
Hall symbol: -P 2ybc	Cell parameters from 3900 reflections
*a* = 19.6325 (11) Å	θ = 2.2–26.9°
*b* = 10.6101 (6) Å	µ = 5.26 mm^−^^1^
*c* = 7.8859 (5) Å	*T* = 296 K
β = 100.078 (1)°	Block, yellow
*V* = 1617.31 (16) Å^3^	0.21 × 0.17 × 0.09 mm
*Z* = 8	

### Data collection

**Table:**

Bruker APEXII CCD diffractometer	3911 independent reflections
Radiation source: fine-focus sealed tube	2706 reflections with *I* > 2σ(*I*)
graphite	*R*_int_ = 0.025
φ and ω scans	θ_max_ = 28.3°, θ_min_ = 2.1°
Absorption correction: multi-scan (*SADABS*; Bruker, 2008)	*h* = −25→25
*T*_min_ = 0.384, *T*_max_ = 0.620	*k* = −13→14
12968 measured reflections	*l* = −10→10

### Refinement

**Table:**

Refinement on *F*^2^	Primary atom site location: structure-invariant direct methods
Least-squares matrix: full	Secondary atom site location: difference Fourier map
*R*[*F*^2^ > 2σ(*F*^2^)] = 0.035	Hydrogen site location: inferred from neighbouring sites
*wR*(*F*^2^) = 0.087	H-atom parameters constrained
*S* = 1.03	*w* = 1/[σ^2^(*F*_o_^2^) + (0.0372*P*)^2^ + 0.7786*P*] where *P* = (*F*_o_^2^ + 2*F*_c_^2^)/3
3911 reflections	(Δ/σ)_max_ = 0.001
181 parameters	Δρ_max_ = 0.82 e Å^−^^3^
0 restraints	Δρ_min_ = −0.77 e Å^−^^3^

### Special details

**Table:**

Geometry. All e.s.d.'s (except the e.s.d. in the dihedral angle between two l.s. planes) are estimated using the full covariance matrix. The cell e.s.d.'s are taken into account individually in the estimation of e.s.d.'s in distances, angles and torsion angles; correlations between e.s.d.'s in cell parameters are only used when they are defined by crystal symmetry. An approximate (isotropic) treatment of cell e.s.d.'s is used for estimating e.s.d.'s involving l.s. planes.
Refinement. Refinement of *F*^2^ against ALL reflections. The weighted *R*-factor *wR* and goodness of fit *S* are based on *F*^2^, conventional *R*-factors *R* are based on *F*, with *F* set to zero for negative *F*^2^. The threshold expression of *F*^2^ > σ(*F*^2^) is used only for calculating *R*-factors(gt) *etc*. and is not relevant to the choice of reflections for refinement. *R*-factors based on *F*^2^ are statistically about twice as large as those based on *F*, and *R*- factors based on ALL data will be even larger.

### Fractional atomic coordinates and isotropic or equivalent isotropic displacement parameters (Å^2^)

**Table:**

	*x*	*y*	*z*	*U*_iso_*/*U*_eq_	
Br2	0.315314 (16)	0.36346 (4)	0.36641 (5)	0.07035 (13)	
Br1	0.538072 (18)	0.69050 (5)	1.27120 (5)	0.08477 (16)	
S2	−0.04123 (4)	0.26135 (7)	0.39188 (10)	0.05003 (18)	
S1	0.21328 (4)	0.52885 (7)	0.75427 (10)	0.05230 (19)	
N2	−0.03383 (11)	0.4881 (2)	0.2688 (3)	0.0473 (6)	
H2A	−0.0119	0.5515	0.2367	0.057*	
H2B	−0.0780	0.4916	0.2623	0.057*	
C1	0.23831 (13)	0.6495 (2)	0.8875 (3)	0.0412 (6)	
C9	0.07643 (12)	0.3842 (2)	0.3369 (3)	0.0342 (5)	
C12	0.21855 (13)	0.3743 (3)	0.3549 (3)	0.0429 (6)	
C10	0.10919 (14)	0.4633 (2)	0.2364 (4)	0.0459 (6)	
H10A	0.0831	0.5200	0.1618	0.055*	
C14	0.11668 (14)	0.3004 (3)	0.4448 (3)	0.0474 (7)	
H14A	0.0956	0.2466	0.5131	0.057*	
C5	0.44606 (15)	0.6804 (3)	1.1491 (4)	0.0558 (8)	
C8	0.00020 (13)	0.3859 (2)	0.3280 (3)	0.0370 (5)	
C13	0.18748 (14)	0.2945 (3)	0.4535 (4)	0.0515 (7)	
H13A	0.2137	0.2366	0.5260	0.062*	
C11	0.18020 (15)	0.4589 (3)	0.2457 (4)	0.0498 (7)	
H11A	0.2018	0.5127	0.1785	0.060*	
N1	0.19425 (12)	0.7375 (2)	0.9174 (3)	0.0554 (6)	
H1A	0.2082	0.7985	0.9867	0.067*	
H1B	0.1517	0.7338	0.8675	0.067*	
C2	0.31088 (13)	0.6623 (2)	0.9781 (3)	0.0408 (6)	
C6	0.40650 (16)	0.7873 (3)	1.1197 (4)	0.0622 (8)	
H6A	0.4250	0.8654	1.1563	0.075*	
C3	0.35274 (15)	0.5562 (3)	1.0060 (4)	0.0549 (7)	
H3A	0.3351	0.4783	0.9659	0.066*	
C7	0.33903 (15)	0.7780 (3)	1.0352 (4)	0.0540 (7)	
H7A	0.3119	0.8502	1.0162	0.065*	
C4	0.42000 (16)	0.5644 (3)	1.0919 (4)	0.0639 (9)	
H4A	0.4475	0.4926	1.1110	0.077*	

### Atomic displacement parameters (Å^2^)

**Table:**

	*U*^11^	*U*^22^	*U*^33^	*U*^12^	*U*^13^	*U*^23^
Br2	0.03962 (17)	0.0895 (3)	0.0834 (3)	0.00005 (15)	0.01476 (15)	0.00159 (19)
Br1	0.04511 (19)	0.1196 (4)	0.0842 (3)	−0.00878 (19)	−0.00386 (16)	−0.0108 (2)
S2	0.0434 (4)	0.0390 (4)	0.0685 (5)	−0.0063 (3)	0.0120 (3)	0.0056 (3)
S1	0.0465 (4)	0.0459 (4)	0.0626 (5)	−0.0022 (3)	0.0043 (3)	−0.0081 (3)
N2	0.0398 (12)	0.0358 (12)	0.0667 (15)	0.0016 (9)	0.0101 (11)	0.0030 (11)
C1	0.0427 (14)	0.0402 (14)	0.0415 (14)	0.0002 (11)	0.0096 (11)	0.0055 (11)
C9	0.0400 (13)	0.0271 (12)	0.0358 (13)	−0.0022 (10)	0.0076 (10)	−0.0035 (10)
C12	0.0405 (14)	0.0435 (15)	0.0448 (15)	−0.0022 (12)	0.0078 (11)	−0.0071 (12)
C10	0.0485 (15)	0.0347 (14)	0.0566 (17)	0.0078 (12)	0.0150 (12)	0.0086 (12)
C14	0.0444 (15)	0.0515 (17)	0.0459 (15)	−0.0021 (12)	0.0067 (12)	0.0152 (13)
C5	0.0392 (15)	0.078 (2)	0.0497 (17)	−0.0057 (15)	0.0057 (12)	−0.0030 (15)
C8	0.0428 (13)	0.0305 (13)	0.0375 (13)	−0.0018 (10)	0.0065 (10)	−0.0057 (10)
C13	0.0420 (15)	0.0607 (18)	0.0499 (16)	0.0040 (13)	0.0023 (12)	0.0138 (14)
C11	0.0552 (17)	0.0372 (15)	0.0628 (18)	−0.0003 (13)	0.0261 (14)	0.0071 (13)
N1	0.0442 (13)	0.0528 (15)	0.0659 (16)	0.0090 (11)	0.0004 (11)	−0.0115 (12)
C2	0.0395 (13)	0.0418 (15)	0.0420 (14)	0.0003 (11)	0.0099 (11)	0.0036 (11)
C6	0.0509 (18)	0.063 (2)	0.072 (2)	−0.0100 (15)	0.0088 (15)	−0.0202 (17)
C3	0.0483 (16)	0.0439 (16)	0.069 (2)	−0.0015 (13)	0.0022 (14)	0.0035 (14)
C7	0.0518 (17)	0.0471 (17)	0.0633 (19)	−0.0007 (13)	0.0108 (14)	−0.0083 (14)
C4	0.0477 (17)	0.058 (2)	0.082 (2)	0.0054 (15)	0.0008 (15)	0.0080 (17)

### Geometric parameters (Å, °)

**Table:**

Br2—C12	1.890 (3)	C14—C13	1.381 (4)
Br1—C5	1.896 (3)	C14—H14A	0.9300
S2—C8	1.675 (3)	C5—C6	1.371 (5)
S1—C1	1.674 (3)	C5—C4	1.379 (5)
N2—C8	1.316 (3)	C13—H13A	0.9300
N2—H2A	0.8600	C11—H11A	0.9300
N2—H2B	0.8600	N1—H1A	0.8600
C1—N1	1.322 (3)	N1—H1B	0.8600
C1—C2	1.484 (4)	C2—C3	1.388 (4)
C9—C14	1.380 (3)	C2—C7	1.389 (4)
C9—C10	1.388 (3)	C6—C7	1.378 (4)
C9—C8	1.486 (3)	C6—H6A	0.9300
C12—C13	1.364 (4)	C3—C4	1.377 (4)
C12—C11	1.373 (4)	C3—H3A	0.9300
C10—C11	1.384 (4)	C7—H7A	0.9300
C10—H10A	0.9300	C4—H4A	0.9300
			
C8—N2—H2A	120.0	C12—C13—C14	119.3 (3)
C8—N2—H2B	120.0	C12—C13—H13A	120.3
H2A—N2—H2B	120.0	C14—C13—H13A	120.3
N1—C1—C2	116.9 (2)	C12—C11—C10	119.5 (3)
N1—C1—S1	121.5 (2)	C12—C11—H11A	120.3
C2—C1—S1	121.58 (19)	C10—C11—H11A	120.3
C14—C9—C10	118.0 (2)	C1—N1—H1A	120.0
C14—C9—C8	120.0 (2)	C1—N1—H1B	120.0
C10—C9—C8	122.0 (2)	H1A—N1—H1B	120.0
C13—C12—C11	120.8 (3)	C3—C2—C7	118.3 (3)
C13—C12—Br2	118.7 (2)	C3—C2—C1	119.6 (2)
C11—C12—Br2	120.4 (2)	C7—C2—C1	122.1 (2)
C11—C10—C9	120.8 (2)	C5—C6—C7	119.3 (3)
C11—C10—H10A	119.6	C5—C6—H6A	120.3
C9—C10—H10A	119.6	C7—C6—H6A	120.3
C9—C14—C13	121.5 (2)	C4—C3—C2	121.1 (3)
C9—C14—H14A	119.2	C4—C3—H3A	119.4
C13—C14—H14A	119.2	C2—C3—H3A	119.4
C6—C5—C4	121.1 (3)	C6—C7—C2	121.0 (3)
C6—C5—Br1	120.0 (2)	C6—C7—H7A	119.5
C4—C5—Br1	118.9 (2)	C2—C7—H7A	119.5
N2—C8—C9	118.1 (2)	C3—C4—C5	119.1 (3)
N2—C8—S2	120.9 (2)	C3—C4—H4A	120.5
C9—C8—S2	120.96 (18)	C5—C4—H4A	120.5
			
C3—C1—C2—S1	23.6 (3)	C14—C8—C9—S2	20.5 (3)

### Hydrogen-bond geometry (Å, °)

**Table:**

*D*—H···*A*	*D*—H	H···*A*	*D*···*A*	*D*—H···*A*
N2—H2A···S2^i^	0.86	2.73	3.583 (2)	172
N2—H2B···S1^ii^	0.86	2.65	3.500 (2)	173
N1—H1A···S1^iii^	0.86	2.78	3.605 (3)	160
N1—H1B···S2^ii^	0.86	2.71	3.523 (2)	158

Symmetry codes: (i) −*x*, *y*+1/2, −*z*+1/2; (ii) −*x*, −*y*+1, −*z*+1; (iii) *x*, −*y*+3/2, *z*+1/2.
